# Identification and characterization of *CsSRP43*, a major gene controlling leaf yellowing in cucumber

**DOI:** 10.1093/hr/uhac212

**Published:** 2022-12-01

**Authors:** Tingting Zhang, Xiangyu Dong, Xin Yuan, Yuanyuan Hong, Lingling Zhang, Xuan Zhang, Shuxia Chen

**Affiliations:** College of Horticulture, Northwest A&F University, Yangling 712100, Shaanxi, China; Shaanxi Engineering Research Center for Vegetables, Yangling 712100, China; College of Horticulture, Northwest A&F University, Yangling 712100, Shaanxi, China; Shaanxi Engineering Research Center for Vegetables, Yangling 712100, China; College of Horticulture, Northwest A&F University, Yangling 712100, Shaanxi, China; Shaanxi Engineering Research Center for Vegetables, Yangling 712100, China; College of Horticulture, Northwest A&F University, Yangling 712100, Shaanxi, China; Shaanxi Engineering Research Center for Vegetables, Yangling 712100, China; College of Horticulture, Northwest A&F University, Yangling 712100, Shaanxi, China; Shaanxi Engineering Research Center for Vegetables, Yangling 712100, China; College of Horticulture, Northwest A&F University, Yangling 712100, Shaanxi, China; Shaanxi Engineering Research Center for Vegetables, Yangling 712100, China; College of Horticulture, Northwest A&F University, Yangling 712100, Shaanxi, China; Shaanxi Engineering Research Center for Vegetables, Yangling 712100, China

## Abstract

Mutants are crucial to extending our understanding of genes and their functions in higher plants. In this study a spontaneous cucumber mutant, *yf*, showed yellow color leaves, had significant decreases in related physiological indexes of photosynthesis characteristics, and had more abnormal chloroplasts and thylakoids. Inheritance analysis indicated that the yellow color of the leaf was controlled by a recessive nuclear locus, *yf*. A candidate gene, *CsSRP43*, encoding a chloroplast signal recognition particle 43 protein, was identified through map-based cloning and whole-genome sequence analysis. Alignment of the *CsSRP43* gene homologs between both parental lines revealed a 7-kb deletion mutation including the promoter region and the coding sequence in the *yf* mutant. In order to determine if the *CsSRP43* gene was involved in the formation of leaf color, the CRISPR/Cas9-mediate system was used to modify *CsSRP43* in the 9930 background; two independent transgenic lines, *srp43-1* and *srp43-2*, were generated, and they showed yellow leaves with abnormal chloroplasts and thylakoids. Transcriptomic analysis revealed that differentially expressed genes associated with the photosynthesis-related pathway were highly enriched between *srp43-1* and wild type, most of which were significantly downregulated in line *srp43-1*. Furthermore, yeast two-hybrid and biomolecular fluorescence complementation assays were used to confirm that CsSRP43 directly interacted with LHCP and cpSRP54 proteins. A model was established to explain the molecular mechanisms by which CsSRP43 participates in the leaf color and photosynthesis pathway, and it provides a valuable basis for understanding the molecular and genetic mechanisms of leaf color in cucumber.

## Introduction

Leaves are important organs for photosynthesis, nutrient transformation, and respiration of plants in nature [71], and photosynthesis is responsible for conversion of light energy and the accumulation of dry matter, so it is very important for the maintenance of normal life activities [69]. The chloroplast is the important organelle responsible for photosynthesis [64], and chlorophyll (Chl) content is one of the most critical factors for photosynthesis; both of them are closely associated with the photosynthetic potential and primary productivity of plants [7, 15, 51]. Leaf color mutants can be used to help us understand the genetic mechanisms of plant photosynthesis, Chl biosynthesis, development, degradation, tetrapyrrole synthesis, and so on [5]. To date, numerous leaf color mutants have been identified and reported in many crops, such as *Arabidopsis* [63], rice [53], soybean [76], wheat [38], and maize [46]. In *Arabidopsis*, nearly 27 genes have been reported to participate in leaf color [71], including *Glu-tRNA* [35], *LHCP* [4], and *FtsH* [3, 41]. In rice, more than 200 leaf color mutants have been reported, and at least 130 related genes have been identified in 12 chromosomes of rice [33, 37]. These genes contribute to chloroplast development [6, 66], chloroplast biosynthesis [72], chloroplast ribosome biosynthesis [16], and other chloroplast functions.

**Figure 1:**
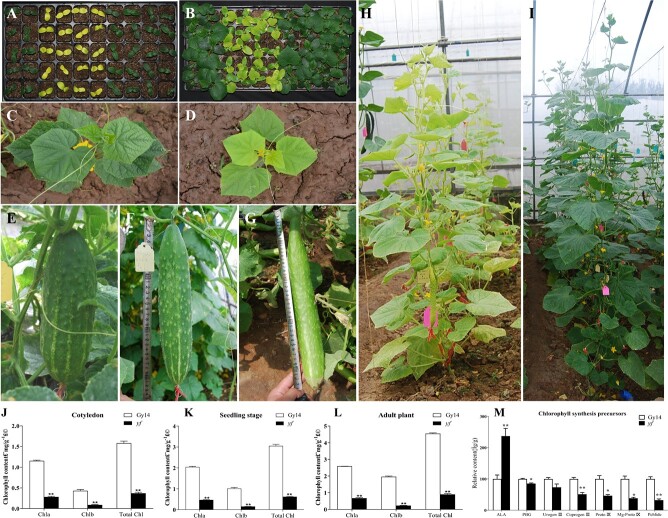
Phenotypic characterization of the *yf* mutant. (A) Wild-type (left), mutant (middle), and *F*_1_ (right) cotyledon stage plants. (B) WT (left), mutant (middle), and *F*_1_ generation (right) two leaves and one heart stage. (C) Gy14 seedling-stage plants. (D) *yf* seedling-stage plants. (E) Gy14 fruit. (F) *F*_1_ adult plant. (G) *yf* fruit. (H) *yf* adult plant. (I) Gy14 adult plant. (J) Chl content of Gy14 and *yf* in the cotyledon stage. (K) Chl content of Gy14 and *yf* in the seedling stage. (L) Chl content of Gy14 and *yf* in the adult plant stage. (M) Chlorophyll precursor content of Gy14 and *yf* in the adult plant stage.

**Figure 2:**
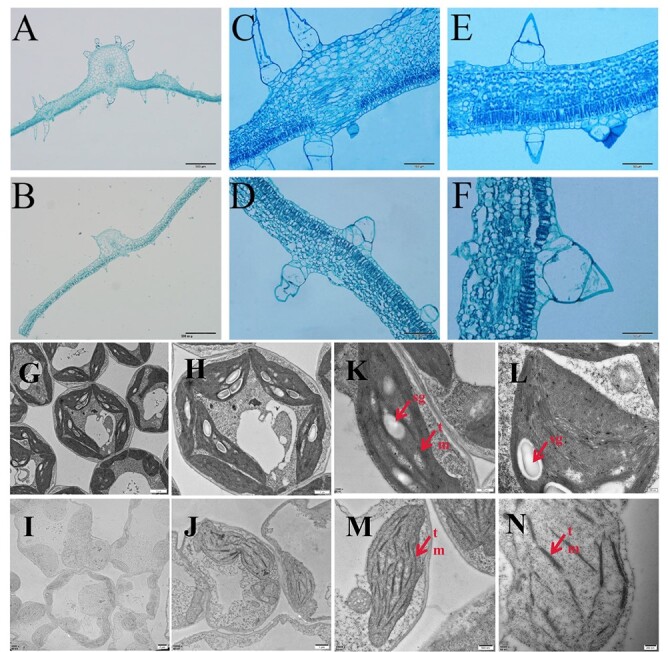
Paraffin sections and ultrastructure of chloroplasts in the yellow mutant and Gy14. (A, C, E) Paraffin-embedded Gy14 leaves. (B, D, F) Paraffin-embedded *yf* leaves. (G–J) Ultrastructure of Gy14 mesophyll cells. (I–N) Ultrastructure of *yf* mesophyll cells. sg, starch grains; tm, thylakoid membranes.

**Figure 3:**
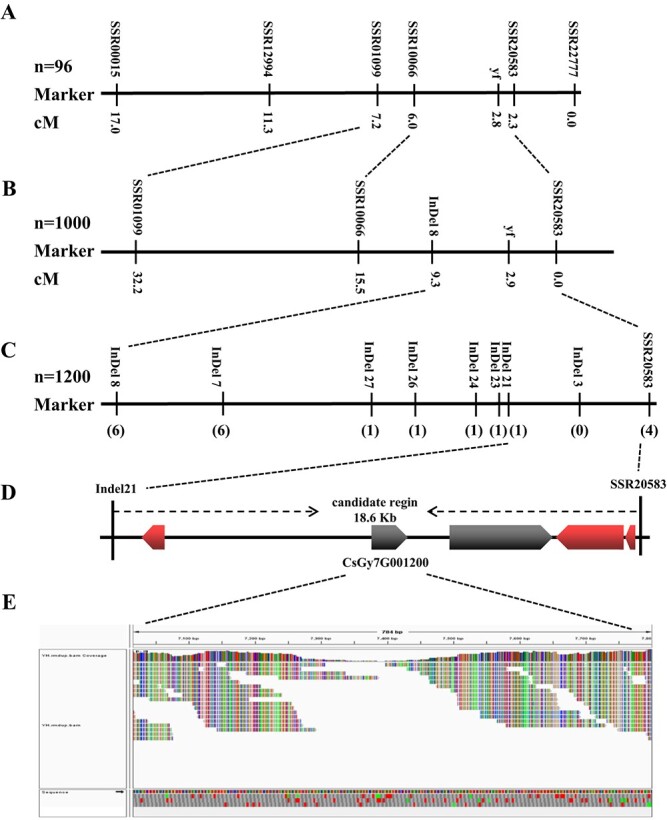
Map-based cloning of the *yf* locus. (A) Initial mapping with 96 *F*_2_ plants placed the *yf* locus between SSR10066 and SSR20583 on cucumber chromosome 7. (B) Further mapping with 1000 *F*_2_ plants placed the *yf* locus between InDel 8 and SSR20583. (C) Fine mapping of the *yf* locus. The numbers in brackets under the marker names indicate the recombinant numbers. (D) Schematic diagram of the five predicted genes. (E) *CsGy7G001220* gene region resequencing analysis.

**Figure 4:**
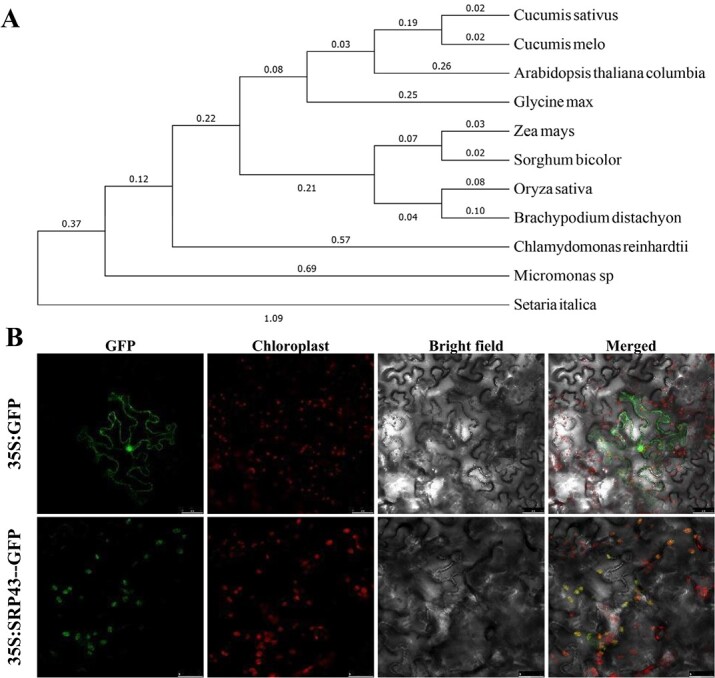
Phylogenetic tree analysis and subcellular localization of the *CsSRP43* in cucumber. (A) Phylogenetic tree of the CsSRP43 protein with 10 homologous proteins from 10 other plant species. (B) Subcellular localization of the CsSRP43:GFP fusion protein.

**Figure 5:**
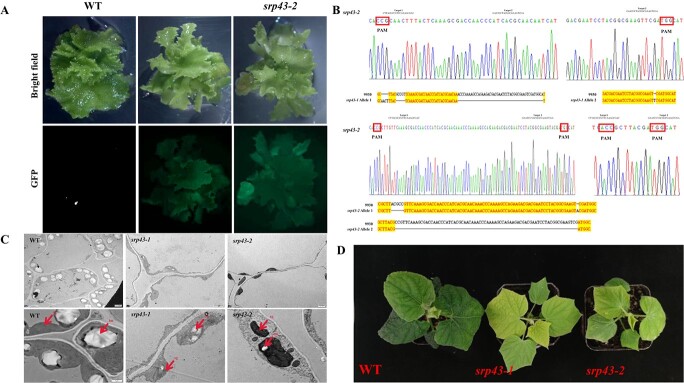
Phenotypic characterization of *CsSRP43* CRISPR/Cas9 transgenic plants. (A) Leaf color and GFP fluorescence of transgenic lines and WT. (B) DNA sequence analysis of targets of *CsSRP43* in 9930, *srp43-1*, and *srp43-2*. (C) Chloroplast ultrastructure in 9930, *srp43-1*, and *srp43-2*. (D) Phenotype of 9930, *srp43-1* and *srp43-2.*

**Figure 6:**
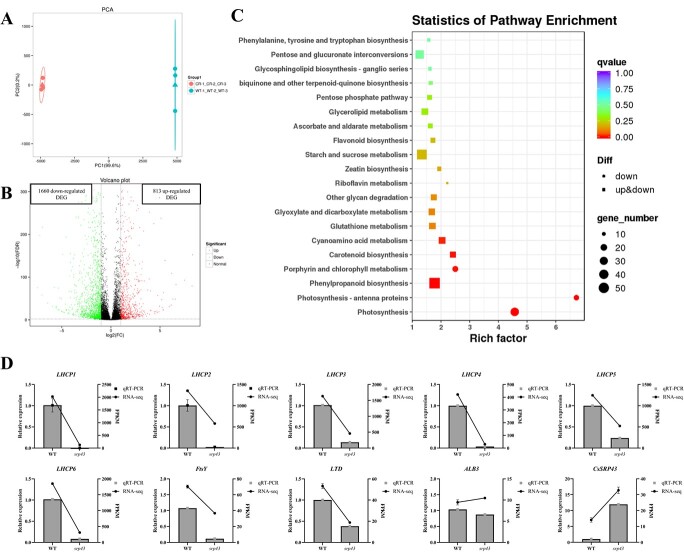
RNA-seq analysis between *srp43-1* and 9930. (A) Principal component analysis analysis between six RNA-seq samples. (B) The number of genes that were upregulated and downregulated between *srp43-1* and 9930. (C) KEGG enrichment analysis of the DEGs. The number of genes is indicated by the size of the bubbles. The adjusted *P* value of the pathways is indicated by the color of the bubbles. (D) RNA-seq results were validated using qRT–PCR.

**Figure 7:**
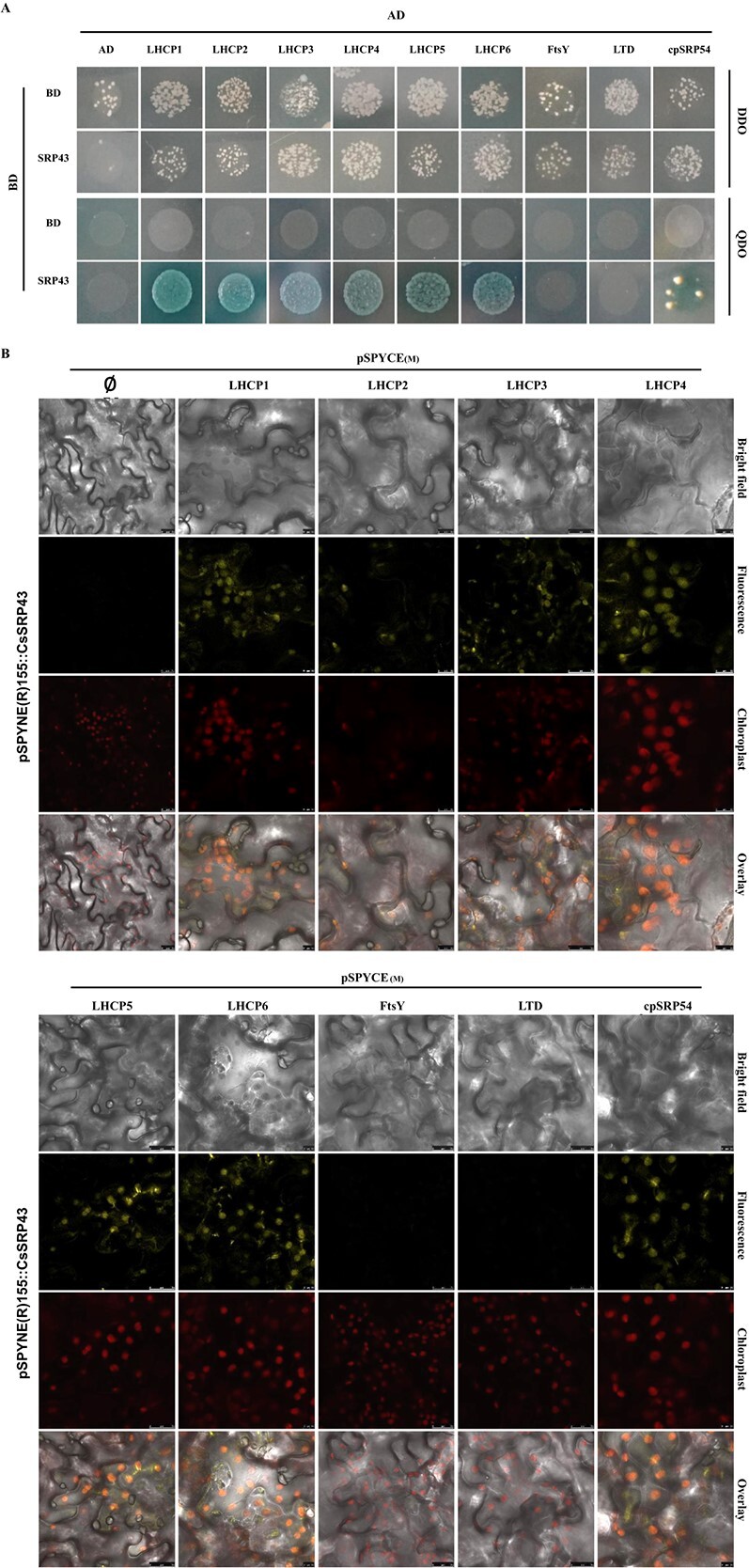
CsSRP43 interacts with SRP54 and LHCP proteins. **(**A) Y2H assay. Yeast cotransformed with constructs or empty vectors (pGADT7 or pGBKT7) as indicated was spotted on no-selection DDO medium and selection QDO medium. (B) BiFC analysis. CsSRP43 as an N-terminal fusion of YFP, and LHCP, FtSY, LTD, or SRP54 as a C-terminal YFP fusion of YFP were coexpressed in *N. benthamiana* leaves. YFP signals were observed by confocal microscope.

Chl is one of the most abundant pigments that determines leaf color in higher plants. It is essential for plants and enables oxygenic photoautotrophs to capture light, transfer excitation energy, and initiate the light reactions of photosynthesis [42]. Chl is synthesized via magnesium (Mg^2+^) chelatase activity, which catalyzes the use of Mg^2+^ for tetrapyrrole biosynthesis [2, 56]. Glutamyl-tRNA reductase (GluTR) is the vital rate-limiting enzyme in tetrapyrrole biosynthesis [43]. Chl functions by binding to various Chl-binding proteins, such as light-harvesting complex proteins (LHCPs) [45]. Newly synthesized Chl functions in the stability, proper folding, and membrane integration of Chl-binding proteins. After translation, LHCP is transported and integrated into the thylakoid membrane by the chloroplast signal recognition particle (cpSRP) pathway transit complex [23, 52]. The CpSRP transit complex consists of cpSRP54 and cpSRP43 and imported LHCP [74].

Cucumber (*Cucumis sativus* L.) is an important fruit vegetable widely cultivated globally. Some leaf color mutants have been discovered in cucumber, but only six genes have been cloned [48]. Miao *et al*. [40] reported a recessive virescent leaf color mutant and identified *CsCNGC*, which encodes a cyclic nucleotide-gated ion channel protein, as the candidate gene in cucumber located on chromosome 6. Gao *et al*. [13] reported a golden leaf mutant in C528 and identified *CsChlI* as the candidate gene involved in chloroplast biosynthesis. Song *et al*. [54] reported a virescent yellow leaf mutant due to a single-nucleotide polymorphism (SNP) mutation in the *CsVYL* gene. This gene encodes a DnaJ-like zinc finger protein that has essential functions in plastid development [54]. A recessive yellow–green leaf mutant (*ygl1*) has been identified, and four tandem *13-LOX* genes are considered candidate genes [10]. A mutant of *yellow young leaf-1* (*yyl-1*) has been identified, and *CsHD*, which encodes an HD domain-containing protein, was considered the candidate gene [20]. Another yellow leaf mutant, *yellow leaf 2.1* (*yl2.1*), was reported, and *pdTPI* was considered the candidate gene, which encodes a plastid isoform of triosephosphate isomerase (pdTPI) protein with important functions in chloroplast biosynthesis [67]. Although these genes causing leaf color changes have been cloned in cucumber, the molecular basis of leaf color remains largely unknown.

In this study, we identified a new yellow leaf color mutant, named *yf*, which was naturally derived from cucumber. Here, we identified locus *yf* by map-based cloning and whole-genome sequencing. The results showed a 7-kb deletion mutation in the cucumber *CsSRP43* gene coding sequence (CDS) and promoter region underlying the mutant phenotype. Consistent with the role of *CsSRP43* in *yf*, CRISPR/Cas9 gene-editing mutations in *CsSRP43* showed abnormal chloroplast development and yellow leaf color. RNA-seq analysis verified that *CsSRP43* influenced the photosynthesis and chlorophyll synthesis pathways. Furthermore, CsSRP43 could directly interact with LHCP and cpSRP54 proteins. This study identified *CsSRP43* function in cucumbers and can help us understand the mechanisms of photosynthesis.

## Results

### Phenotypic characterizations of *yf* mutants in cucumber

The *yf* mutant, a naturally derived mutant isolated from an unknown cucumber line, showed a yellow leaf phenotype. Compared with Gy14, which has a normal green leaf color, the *yf* mutant displayed a yellow leaf color throughout the cotyledon, seedling, and adult plant developmental stages (Fig. 1A–G). Furthermore, the *yf* mutant was slightly smaller than the wild-type (WT) plant (Fig. 1A–G, Supplementary Data Table S1).

Chl and Chl precursors were measured to investigate whether the photosynthetic apparatus was influenced by the *yf* mutant. We compared Chla, Chlb, and total Chl contents between *yf* and Gy14 at the cotyledon stage (Fig. 1A), seedling stage (Fig. 1C, 1D), and adult plant stage (Fig. 1H, 1I). Chla, Chlb, and total Chl in *yf* were all significantly lower than those in Gy14, which were 24.28%, 20.82%, and 23.34% in the cotyledon stage, 22.96%, 15.23%, and 20.40% in the seedling stage, and 26.01%, 11.56%, and 19.79% in the adult plant stage, respectively. For the Chl precursors, porphobilinogen (PBG), coproporphyrinogen III (coprogen III), protoporphyrin III (Proto IX), Mg-protoporphyrin IX (Mg-Proto IX), and protochlorophyllide (Pchlide) were significantly decreased compared with Gy14 (Fig. 1L). However, 5-aminolevulinic acid (ALA) was 2.37-fold higher in *yf* than Gy14. These results suggested that the *yf* mutant’s yellow leaf color was probably due to the reduction of Chl caused by a decrease in conversion of ALA into PBG.

The photosynthetic parameters and chlorophyll fluorescence kinetics were measured to investigate whether photosynthesis was affected in *yf* (Supplementary Data Table S2). The results showed that net photosynthesis (Pn) was significantly lower in *yf* than in Gy14. Pn in *yf* was 17.4% in the seedling stage and 80.5% in the adult plant stage of Gy14. The intracellular CO_2_ concentration (Ci) in *yf* was significantly higher than that in Gy14, indicating that *yf* had a reduction in CO_2_ consumption. For chlorophyll fluorescence, the initial fluorescence (F0), maximum fluorescence (Fm), maximum quantum efficiency of photosystem II (Fv/Fm), photochemical quenching (qP), and non-photochemical quenching (qN) in *yf* were significantly lower than in Gy14 (Supplementary Data Table S3). These observations indicated that Chl deficiency in *yf* results in the inability to convert light quanta into chemical energy.

We examined the cells of leaves for Gy14 and *yf* true leaves, as shown in Fig. 2A–F. Compared with Gy14, the palisade organization of the leaves in *yf* was scattered, and the second layer of palisade tissue cells was reduced. Further, spongy tissue was scattered in *yf* compared with Gy14. To investigate how the *yf* mutation affected chloroplast development, we examined the ultrastructure of chloroplasts of Gy14 and *yf* leaves by transmission electron microscopy (TEM). Compared with Gy14, abnormal chloroplasts were found in *yf* leaves (Fig. 2G–N). A decrease in thylakoids and starch granules in the mesophyll cells was observed in *yf*. There was no significant difference in the number of chloroplasts between GY14 and *yf* (Supplementary Data Fig. S1). These observations indicated that chloroplast development was defective in the *yf* mutant.

**Table 1:** Genetic inheritance analysis of different generations of the Gy14 and *yf* plants.

Material	Total population	Green plants	Yellow plants	Expected ratio	χ^2^ Value
Gy14	15	15	0	1:0	
*yf*	15	0	15	0:1	
F1	30	30	0	1:0	
F2 (*yf*×Gy14)	593	452	141	3:1	0.261

### Fine mapping and identification of the candidate gene for *yf*

For the genetic analysis of the *yf* mutant, *yf* was used as the male parent in a cross with Gy14, resulting in *F*_1_ and *F*_2_ populations. All *F*_1_ plants from the crosses displayed a green leaf color phenotype. Among the 593 *F*_2_ individuals, 141 plants showed yellow leaf color and 452 normal green leaf color, which fitted well to the expected 1:3 segregation ratio (Table 1). These results suggested that yellow leaf color is controlled by a single recessive nuclear gene underlying the *yf* mutation.

Using 900 simple sequence repeat (SSR) markers, 247 (27.4%) were polymorphic between *yf* and Gy14. Six of the 247 SSR markers were polymorphic between Gy14 and *yf*, including SSR00015, SSR10066, SSR22777, SSR20583, SSR12994, and SSR01099, and all of these were located on cucumber chromosome 7 (Supplementary Data Table S7). Linkage analysis of 96 *F*_2_ individuals developed from Gy14 × *yf* with the six SSR markers indicated that the *yf* locus was located within a 4.9-cM interval between SSR10066 and SSR20583 (Fig. 3A). Then, we added 1000 plants to the *F*_2_ population. Meanwhile, whole-genome sequencing of *yf* was performed to find new polymorphic markers between Gy14 and *yf*. Thus, 13 InDel markers were tested for polymorphism and one new InDel marker, InDel8, between SSR20583 and SSR10066 was identified. New linkage analysis with 1000 *F*_2_ seedlings and four markers was conducted, and the *yf* locus was found to be located between InDel8 and SSR20258 (Fig. 3B). Another eight recombinants were obtained after screening 1200 *F*_2_ seedlings with InDel8 and SSR20258. Seven new polymorphic InDel markers were used to genotype these eight recombinants for precise mapping. The *yf* locus was further narrowed to an 18.6-kb region between InDel21 and SSR20258, with one and four recombinants (Fig. 3C).

According to the cucumber Gy14 reference annotation file, there were five predicted genes in this 18.6-kb region (Fig. 3D). These annotated genes mainly consist of l-ascorbate oxidase homolog, signal recognition particle 43 kDa protein (cpSRP43), transmembrane protein, and two lysine histidine transporter-like 8 genes. The annotations of the five genes is shown in Supplementary Data Table S4. To identify a possible *yf* locus candidate gene, we analyzed the expression patterns of the five genes in leaves between Gy14 and *yf*. The expression of *CsGy7G001200*, *CsGy7G001240*, and *CsGy7G001250* showed slight differences between Gy14 and *yf*, and *CsGy7G001230* had increased expression in *yf* compared with Gy14. However, *CsGy7G001220* was significantly downregulated in *yf* compared with Gy14 (Supplementary Data Fig. S2). According to the whole-genome resequencing data of *CsGy7G001230*, there were only two lowly reliable synonymous SNPs in the exon region between *yf* and Gy14. However, whole-genome sequence analysis revealed that the *CsGy7G001220* gene and its promoter region had a 7-kb deletion in *yf*, which was predicted to encode the cpSRP43protein (Fig. 3E). Based on the expression patterns, whole-genome sequence analysis and functional annotation of these genes, we identified *CsGy7G001220* as the most likely candidate gene for locus *yf*.

### Analysis of the putative candidate gene for *CsSRP43*

To better understand the genetic and functional differences in CsSRP43 between cucumber and other species, 10 reported cpSRP43 orthologous proteins in other species were analyzed. A phylogenetic tree was constructed, and the results showed that CsSRP43 had the highest percentage of identity with the orthologous protein in melon (Fig. 4A). Furthermore, the phylogenetic tree indicated that cpSRP43 proteins from higher plants can be placed in one subgroup that is distinct from cpSRP43 proteins found in lower plants. To detect the subcellular localization of CsSRP43, we performed a transient expression assay using tobacco leaves. Green fluorescent protein (GFP) fluorescence signals showed that the CsSRP43:GFP fusion protein was colocalized with Chl autofluorescence signals in chloroplasts (Fig. 4B). These results demonstrated that *CsSRP43* was targeted to cucumber chloroplasts..

### Mutagenesis of *CsSRP43* resulted in a yellow leaf color phenotype

To further verify if the *CsSRP43* gene controlled leaf yellowing in cucumber, we generated two independent transgenic lines, *srp43-1* and *srp43-2*, using CRISPR/Cas9 in the 9930 background (Fig. 5A). As expected, yellow leaves were observed in the *srp43-1* and *srp43-2* lines compared with the WT green leaf color. Lines *srp43-1* and *srp43-2* had different gene-edited versions of *CsSRP43*. In *srp43-1*, allele 1 had seven SNPs in target 1 and a 46-bp deletion in target 2, while allele 2 had a 1-bp insertion in target 2. In *srp43-2,* allele 1 had a 5-bp deletion in target 1 and a 1-bp insertion in target 2, while allele 2 had a 72-bp deletion between target 1 and target 2 (Fig. 5B). Abnormal chloroplasts in the leaves, fewer chloroplast thylakoids, and smaller starch granules in the mesophyll cells of *srp43-1* and *srp43-2* were found by chloroplast ultrastructure observation by TEM, compared with 9930 (Fig. 5C). These results indicated that *CsSRP43* was the gene controlling yellow leaf color in cucumber. Because *srp43-1* and *srp43-2* had a similar phenotype with mutant *yf*, we chose the *srp43-1* transgenic line for further transcriptomic analysis (Fig. 5D).

### *CsSRP43* regulated transcription of multiple genes associated with photosynthesis

To investigate the molecular mechanism of *CsSRP43* in the regulation of leaf yellowing, we collected the second true leaves of 9930 (WT) and *srp43-1* to perform RNA-seq with three biological replicates, named WT-1, WT-2, WT-3, CR-1, CR-2, and CR-3. Approximately 25 million clean reads were generated for each sample, and ~93% of clean reads had Phred-like quality scores that reached the Q30 level in the six samples (Supplementary Data Table S5). Principal component analysis showed that the sequencing data were highly reproducible between all six samples, which indicated that the RNA-seq data were reliable (Fig. 6A). There were 2473 differentially expressed genes (DEGs) (*P*_adj_ ≤.05; |log_2_(fold change)| ≥1), of which 813 were downregulated and 1660 were upregulated in CR compared with WT (Fig. 6B).

Gene Ontology (GO) term enrichment was used to investigate the functions of the DEGs between *srp43-1* and WT. In the cellular component category, the DEGs were significantly enriched in chloroplast thylakoid membrane (GO:0009535), chloroplast (GO:0009507), and photosystem I (GO:0009522). Among the molecular function categories, the DEGs were significantly enriched in chlorophyll binding (GO:0016168), heme binding (GO:0020037), and iron ion binding (GO:0005506). Among the biological process category, the DEGs were significantly enriched in photosynthesis (GO:0015979), photosynthesis, light harvesting in photosystem I (GO:0009768), and protein-chromophore linkage (GO:0018298) (Supplementary Data Fig. S3). All the most significant enrichment terms were associated with photosynthesis.

Kyoto Encyclopedia of Genes and Genomes (KEGG) analysis showed that the differences in gene transcript abundance between *srp43-1* and WT leaves led to the identification of 121 enriched pathways. However, the top five enriched KEGG pathways were photosynthesis (ko00195), photosynthesis-antenna proteins (ko00196), phenylpropanoid biosynthesis (ko00940), porphyrin and chlorophyll metabolism (ko00860), and carotenoid biosynthesis (ko00906) (Fig. 6C, Supplementary Data Table S6). All the genes in the photosynthesis, photosynthesis-antenna proteins, and porphyrin and chlorophyll metabolism pathways and most of the genes in the phenylpropanoid biosynthesis and carotenoid biosynthesis pathways were downregulated in the *srp43-1* line compared with WT. The DEGs in these pathways were related to genes involved in photosystem I, photosystem II, cytochrome b6/f complex, photosynthetic electron transport, F-type ATPase, light-harvesting chlorophyll protein complex (*LHCP*) and so on.

To verify the accuracy of the RNA-seq data in this study, 10 DEGs (six *LHCP* genes, one *FtsY* gene, one *LTD* gene, one *ALB3* gene, and *CsSRP43*) related to the photosynthesis pathway were selected to measure relative transcription via qRT–PCR analysis (Fig. 6D). The results of qRT–PCR for the 10 selected genes were consistent with the RNA-seq data, suggesting that the DEGs in the RNA-seq analysis were reliable.

### CsSRP43 interacted with cpSRP54 and LHCP proteins

In previous studies, the cpSRP54, LHCP, FtsY, and LTD proteins were shown to be involved in chloroplast cpSRP pathways [68]; thus, a yeast two-hybrid (Y2H) assay was used to examine the protein–protein interactions between CsSRP43 and LHCP1 (CsaV3_6G051530), LHCP2 (CsaV3_6G051520), LHCP3 (CsaV3_3G031580), LHCP4 (CsaV3_1G032510), LHCP5 (CsaV3_7G002620), LHCP6 (CsaV3_5G039350), FtsY (CsaV3_3G009150), LTD (CsaV3_3G005280), and cpSRP54 (CsaV3_4G001950). The results showed that CsSRP43 interacted with the six LHCP proteins and cpSRP54 but not with FtsY and LTD (Fig. 7A). To further validate the interactions between CsSRP43 and interaction components, a biomolecular fluorescence complementation (BiFC) assay was conducted; it confirmed the interactions between CsSRP43 and the six LHCP proteins, and the interaction between CsSRP43 and SPR54 was also confirmed (Fig. 7B).

## Discussion

### The *CsSRP43* gene causes changes in chlorophyll content and photoreaction stability during photosynthesis in cucumber

In plants, Chl was an essential pigment in the photoreaction that absorbs solar energy and binds various Chl-binding proteins to transfer and transform energy. CpSRP43 was a chaperone for glutamyl-tRNA reductase, which was the rate-limiting enzyme for tetrapyrrole biosynthesis. Tetrapyrrole, together with glutamate 1-semialdehyde aminotransferase (GSAAT), synthesizes ALA, which was one of the precursors for Chl synthesis [62]. Furthermore, cpSRP43 maintained and protected Chl biosynthesis under heat shock conditions in *Arabidopsis* [22].

The Y2H and BiFC assays in this study showed that CsSRP43 is a cpSRP54 chaperone and, combined with LHCP, might function in the delivery of LHCP to the thylakoid membrane, as previously reported in other species [11]. In cucumber, disruption of the LHCP protein directly or indirectly reduced fluorescence intensity in the photosynthetic system [58]. This might be the reason why the chlorophyll fluorescence of *yf* was significantly lower than that of Gy14. In addition, the Chla, Chlb, and total Chl contents in *yf* leaves were all significantly lower than those in Gy14 leaves, and the amount converted from ALA to PBG was reduced. Furthermore, the KEGG analysis for RNA-seq showed that porphyrin and chlorophyll metabolism pathway-related genes were enriched in the *srp43-1* line and all of these genes were downregulated compared with WT. This result further verified that *CsSRP43* participates in chlorophyll synthesis, which also explained the reduced starch granules in chloroplasts and the smaller plant size phenotype observed in the *yf* mutant. However, further evidence of *CsSRP43* function in Chl biosynthesis is lacking and needs to be explored in cucumber.

### The phenotype of abnormal chloroplast and thylakoid structures in the *CsSRP43* mutant might cause substantial decreases in LHCP

To date, six genes that control leaf color in cucumber have been cloned. Most of these mutants showed changes in the number or structure of chloroplasts and thylakoids in leaves [54, 67]. However, none of these reports have explored or explained this phenotype. In this study, *CsSRP43* gene deficiency caused a yellow leaf color phenotype with abnormal chloroplasts and thylakoids in cucumber leaves. This finding indicated that *CsSRP43* also participated in chloroplast and thylakoid development.

Abnormal thylakoid structures were also observed in *cpFtsY*, *Alb3*, and double mutants of *cpSRP43* and *cpSRP54* in *Arabidopsis* [12, 21, 57]. In particular, all of these genes participated in LHCP targeting from the stroma to the thylakoid membrane, and cpSRP was the predominant target in this process [75]. The double mutant (*cpSRP43* and *cpSRP54*) showed a near-total loss of LHCP protein in the thylakoid membrane in *Arabidopsis*. Thus, the abnormal chloroplast and thylakoid structures affected by the CsSRP43 mutant probably caused the substantial decreases in LHCP. However, the mutant of *cpSRP43* in *Arabidopsis* showed no differences in chloroplasts from the WT [24]. This indicated that *CsSRP43* might have a different function from *cpSRP43* in *Arabidopsis*.

### *CsSRP43* is a newly identified yellow leaf gene and an important component of cucumber chloroplast cpSRP complex

To date, the genes that control leaf color in cucumbers have been reported to include *CsCNGCs* [40], *CsChlI* [13], *CsVYL* [54], *LOXs* [10], *CsHD* [20], and *pdTPI* [67]. These genes were involved in chloroplast biosynthesis, plastid development, or are functionally unknown. Furthermore, seven genes that controlled cucumber peel color have been reported [28, 32, 36, 47, 65, 70, 73]. These genes were associated with chlorophyll biosynthesis, and two of these genes were MYB transcription factors. In this study, we identified a new gene, *CsSRP43*, which controlled the yellow color of cucumber leaves. Deletion of the *CsSRP43* CDS and its promoter region resulted in yellowing of cucumber leaves. The importance of the *CsSRP43* gene for cucumber leaf color was further demonstrated by directed mutation using the CRISPR/CAS9 system. However, the exploration of *CsSRP43* promoter function was lacking. Variation in the promoter led to changes in plant phenotype, which indicated the importance of plant promoter segments for their gene function [25, 60]. This might be due to the *cis*-acting elements of the target gene promoters controlling the expression levels of related genes [32]. Therefore, the promoter fragment of *CsSRP43* might also play an important role in cucumber leaf color.

**Figure 8:**
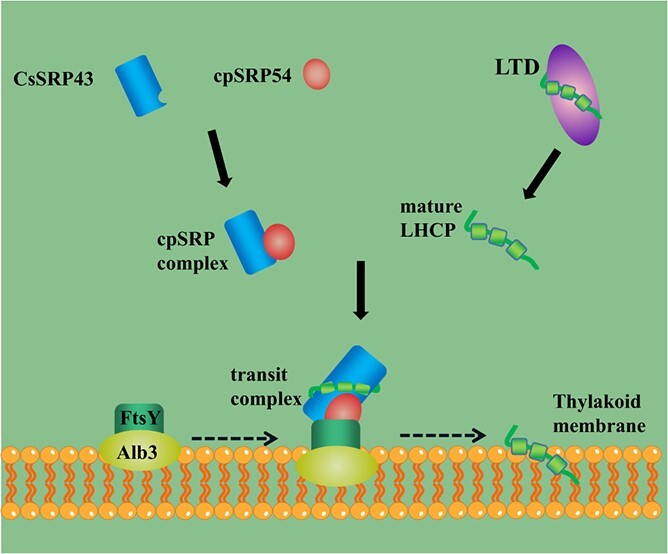
Putative working model of the regulatory function of *CsSRP43* in cucumber leaf color. CsSRP43 interacts with cpSRP54 to form the cpSRP complex, and then the mature LHCP proteins are transferred to the cpSRP complex by chloroplast stromal protein LTD and interact with CsSRP43. With the combined cpSRP complex, LHCP protein transfers into the thylakoid membrane in a process that requires FtsY and Alb3.

### A model was established to explain the molecular mechanisms by which *CsSRP43* participates in the yellow leaf color and photosynthesis pathway

We propose that the *CsSRP43* gene is involved in chlorophyll biosynthesis and photosystem I and II systems based on phenotypic observation, transcriptome analysis, and verification of protein–protein interactions. However, the detailed function of *CsSRP43* still needs to be explored. *CpSRP43* was a highly conserved functional gene that exists only in plants, so the current research results of *cpSRP43* in other species can provide information for *CsSRP43*. The conserved domain of CsSRP43 consisted of four ankyrin repeats and three chromodomains (CD_CDS). The same conserved domain was also observed in *CAO*, a homolog of CsSRP43 in *Arabidopsis*. CpSRP43 interacted with cpSRP54 to form the cpSRP complex mainly by the interaction of one of the CD2 chromodomains with the ARRKR motif in cpSRP54 [18]. The ankyrin repeats in the cpSRP43 protein indicated that the cpSRP43 interaction with LHCP involves binding to an L18 motif and a hydrophobic region in the LHCP protein [9, 55]. As a receptor for the cpSRP complex, cpFtsY bound to thylakoid membranes and linked to LHCP protein insertion via interactions with cpSRP54 [39]. According to this study’s results, CsSRP43 cannot interact with cpFtsY. These findings provided insights for further studies into the molecular mechanism of *CsSRP43*.

On the basis of transcriptomic analysis and Y2H assay and BiFC conformation results, combined with current knowledge about *cpSRP43* in other species, we propose a putative model for *CsSRP43*, which played an important role in leaf color and participated in the photosynthesis pathway in cucumber (Fig. 8). In this model, CsSRP43 interacts with cpSRP54 to form the cpSRP complex, and then the mature LHCP proteins are transferred to the cpSRP complex by chloroplast stromal protein LTD and interact with CsSRP43. With the combined cpSRP complex, LHCP protein transfers into the thylakoid membrane in a process that requires FtsY and Alb3. So CsSRP43 is one of the important components participating in the formation of the cpSRP complex, receiving LHCP proteins, and conducting the signals of photosynthesis pathways. In the mutant of *yf*, the complex cannot form and photosynthesis pathway transmission is affected, which might be the reason for the yellow leaf color.

## Materials and methods

### Plant materials, growth conditions, and phenotypic data collection

The yellow leaf color mutant (*yf*) was naturally derived from an unknown cucumber line. To map the yellow leaf color for *yf*, we constructed the *F*_1_ and *F*_2_ segregation populations by crossing *yf* with the inbred Gy14 line, which has green cotyledons and leaves. Another North China-type inbred line (9930, also known as China Long) with green leaves was used as transgenic material for mutation of the *yf* candidate gene using the CRISPR/Cas9 system. All the materials described above were planted in a plastic tunnel at the Horticulture Experimental Station (34°16′ N, 108°4′ E) of the College of Horticulture, Northwest A&F University, Yangling, Shaanxi Province, China. The row spacing was 60 cm and the plant spacing was 25 cm.

The cucumber leaf color was determined by visual inspection on a sunny day. Plant height was measured using a soft measuring tape, and the stem diameter was measured using a digital caliper when the plant had grown to the final adult stage. The leaves of the 13th, 14th, and 15th true leaves were photographed, and ImageJ software (National Institutes of Health, USA) was used to measure leaf length, leaf diameter, and leaf area. Three plants from different lines were used for every biological replicate, and the experiment was repeated three times.

### Chlorophyll and Chlorophyll precursor content measurements

The contents of Chla and Chlb for the mutants *yf* and Gy14 were measured at the cotyledon stage, seeding stage, and adult plant stage, respectively. The cotyledon at the cotyledon stage, the second true leaf when the plant had grown to the three or four true leaf stage, and 14 true leaves of the adult plant stage were collected for chlorophyll measurements. Three plants from each line were used as the mixture for every biological replicate, and the experiment was repeated three times. Cotyledons or leaf samples (0.2 g) were extracted with 20 ml of extraction solution (ethanol:acetone:water = 4.5:4.5:1) and kept in the dark for 24 hours at room temperature. Finally, the extract was measured using a WFZ UV-3802H spectrophotometer at 663, 645, and 470 nm. The detailed method followed that described in Lichtenthaler *et al*. [26].

ALA, PBG, Proto III, coprogen III, Proto IX, Mg-Proto IX, and Pchlide were used as the chlorophyll precursors and measured for the 13th, 14th, and 15th true leaves at the complete adult plant stage. Three plants from different lines were used for every biological replicate, and the experiment was repeated three times. ALA was measured according to the method described by Dei *et al*. [8]. PBG, Proto III, and coprogen III were measured according to Bogorad *et al*. [1]. Proto IX, Mg-Proto IX, and Pchlide were measured according to [29].

### Photosynthetic parameters and chlorophyll fluorescence kinetic measurements

The photosynthetic parameters and chlorophyll fluorescence were measured at 11:00 in the morning on a sunny day. The 13th, 14th, and 15th true leaves were selected and measured. Three plants were measured for every biological replicate, and the experiment was repeated three times. The photosynthetic parameters, including Pn, stomatal conductance (Gs), Ci, and transpiration rate (Tr), were measured with a photosynthetic instrument (Li-6400XT, Li-Cor Ltd, USA). The detailed methods were described by Liu *et al*. [27].

The chlorophyll fluorescence kinetic measurements included F0, Fm, Fv/Fm, qP, effective photochemical quantum yield of (Y II), and qN. Chlorophyll fluorescence was measured using a pulse-modulated fluorometer (PAM-2500, Germany). The detailed measurements were performed according to Ghorbanpour *et al*. [14].

### Mapping strategy, molecular marker development, and candidate gene identification of *yf* locus

Bulked segregation analysis (BSA) was used to identify the markers linked to the *yf* locus. Genomic DNA was extracted using the CTAB method, according to Murray and Thompson [44]. Gy14 and *yf* bulked DNA samples were constructed by mixing 15 plants from the *F*_2_ population. Polymorphic SSR markers between Gy14 and *yf* were identified and applied to the two bulk DNA samples. The *yf* locus was primarily mapped using 96 *F*_2_ individuals. Linkage analysis for the *yf* locus and the molecular markers was performed with JoinMap 4.0 software, applying a 4.0 LOD threshold value. When no recombination lines were observed between the flanking markers, a larger *F*_2_ population (*n* = 1000, including the initial 96 plants) was used for further testing. For fine mapping, whole-genome sequencing for *yf* was performed, and the sequence reads of *yf* cucumber lines were aligned with Integrative Genomics Viewer (IGV) software using the Gy14 draft genome as reference. A total of eight InDel markers were identified, and the *F*_2_ population was expanded to 1200 plants (including the initial 1000 plants) to fine map the *yf* locus. InDel markers were designed by Primer 5.0 software, and the primers used for linkage analysis are listed in Supplementary Data Table S7.

Candidate gene prediction was based on the genome database of cucumber (Gy14) v2 (http://cucurbitgenomics.org/organism/16). Gene function predictions were conducted with BLASTP (http://blast.ncbi.nlm.nih.gov). The primers used for cloning the candidate genes were designed by Primer Premier 5.0 and are listed in Supplementary Data Table S8.

### Multiple-sequence alignment and phylogenetic analysis

Protein sequences of the *yf* candidate gene and its homologs were obtained from the NCBI database. Phylogenetic analysis for CsSRP43 protein with 10 homologous proteins from 10 other plant species was done using MEGA 6.0 software according to Saitou *et al*. [50]. The 10 other proteins utilized were *Arabidopsis thaliana* OAP10661.1, *Cucumis melon* XM_008449677, *Glycine max* (soybean) XP_003537753.1, *Zea mays* (maize) NP_001168899.1, *Sorghum bicolor* XP_002465920.1, *Oryza sativa* (rice) ALJ30353.1, *Brachypodium distachyon* XP_003562136.1, *Chlamydomonas reinhardtii* AGC59877.1, *Micromonas* sp. XP_002507724.1, and *Setaria italica* (millet) XP_004985832.3.

### RNA extraction and quantitative real-time PCR

The second true leaf was collected for RNA extraction when the plants had grown to the three or four true leaf stage. The reference gene *UBQ* was used as an internal control [59, 61]. Total RNA was extracted using TriPure Reagent (DiNing, China). The cDNA fragments were synthesized using the Transcriptor First Strand cDNA Synthesis Kit (Roche, USA). qRT–PCRs were performed using SYBR Green Master Mix (GeneStar, China) with a 20-μl reaction on a QuantStudio5 instrument (Life Technologies, USA), and the data were analyzed using the 2^-ΔΔCT^ method normalized to ubiquitin [34]. The qRT–PCR primer sequences are listed in Supplementary Data Table S8. Reactions were carried out using two biological and four technical replicates for each sample.

### Subcellular localization

The CDS of *CsSRP43* was cloned without the stop codon from Gy14 and fused in-frame into the expression vector pCambia2300-35S-GFP. The construct pCambia2300-35S-*CsSRP43*-GFP was transformed into *Agrobacterium* GV3101 and then infiltrated into tobacco leaves. Empty pCambia2300-35S-GFP vector was used as a control. After 60 hours at 24°C, the epidermis of tobacco was observed under a laser scanning confocal microscope (Leica TCS-SP8 SR, Germany).

### Microscopic observation of leaf cells

Paraffin sections of Gy14 and *yf* leaves were prepared according to Guidarelli *et al*. [17]. The 13th, 14th, and 15th true leaves were collected, cut into 0.5 cm × 0.5 cm pieces, and immediately fixed in 70% FAA solution. The tissues were then transferred into a graded ethanol series (10–100%) and chloroform series (10–100%) and embedded in paraffin. Then, the samples were cut into 8-μm thick sections, stained with safranin, and sealed with gum. The sections were observed under an Olympus BX51 microscope (Olympus, Japan).

### Transmission electron microscopy

The Gy14 and *yf* leaf samples were collected from the 14th true leaves for TEM. Leaf samples of the 9930 WT and *CsSRP43* CRISPR transgenic lines were collected from the second expanded leaf. These leaves were cut into small pieces of ~0.2 mm × 0.2 mm size and fixed in a 4.0% glutaraldehyde solution. Leaf samples were prepared for TEM according to Xiong *et al*. [67]. Finally, the samples were observed using a Tecnai G2 Spirit Bio (FEI, USA) transmission electron microscope.

### Cucumber transformation

Two sgRNA binding sites in the CDS of the *CsSRP43* gene (target 1 and target 2) were selected with the CRISPR-P v2.0 tool (http://cbi.hzau.edu.cn/CRISPR2/) and assembled into the CRISPR/Cas9 vector pBSE402. The primers used in vector construction are listed in Supplementary Data Table S8. Then, the vector was transformed into *Agrobacterium* EHA105. *Agrobacterium tumefaciens* with the *CsSRP43* CRISPR/Cas9 vector was transformed into the cucumber line 9930 by using cotyledonary node transformation as previously described [19]. The regenerated plants were screened by GFP fluorescence and sequencing of the *CsSRP43* gene.

### Transcriptome analysis

Line 9930 and the *CsSRP43* CRISPR/Cas9-edited *srp43-1* line were sampled from the second expanded leaf. RNA-seq was commissioned from the Beijing Biomarker Company to construct a strand-specific library followed by sequencing on an Illumina HiSeq 2500 instrument. The adaptor sequences containing N and low-quality reads were filtered and removed, and the clean reads were mapped to the cucumber (Chinese Long) v3 genome (http://cucurbitgenomics.org/organism/20). To define the significance of DEGs in the RNA-seq results, we set a significance threshold of |log_2_(fold change)| ≥ 1.0 with adjusted *P* value ≤0.05. The gene expression levels were based on the numbers of expected reads per kilobase of transcript sequence per million base pairs sequenced (FPKM). Three leaves of 9930 and *srp43-1* were used as one replicate, and three replicates were included (total of six samples).

### Yeast two-hybrid assay

Y2H assays were performed using the Matchmaker™ Gold Two-Hybrid System (Clontech) following the manufacturer’s instructions. The full-length *CsSRP43* cDNA was inserted into a pGADT7 (AD) vector, while the CDS fragments of *CsaV3_4G001950* (*LHCP1*), *CsaV3_6G051520* (*LHCP2*), *CsaV3_3G031580* (*LHCP3*), *CsaV3_1G032510* (*LHCP4*), *CsaV3_7G002620* (*LHCP5*), *CsaV3_5G039350* (*LHCP6*), *CsaV3_3G005280* (*LTD*), *CsaV3_3G009150* (*FtsY*), and *CsaV3_4G001950* (*cpSRP54*) were inserted into a pGBKTD7 (BD) vector. The primer pairs used for AD and BD vector construction are listed in Supplementary Data Table S8. Combinations of BD and the nine AD vectors were cotransformed into the Y2H Gold strain and plated on −Trp/−Leu (DDO) medium for 3 days at 28°C. Then, the nine interactions were tested on SD–Leu–Trp–His–Ade containing X-α-Gal media plates (QDO).

### Bimolecular fluorescence complementation assay

For the bimolecular fluorescence complementation (BiFC) assay, the *CsSRP43* CDS was amplified using Phanta Super-Fidelity DNA Polymerase Mix (Vazyme, China) and inserted into SPYNE(R)155 vector through BamHI and SmaI sites to produce pSPYNE(R)155::CsSRP43 nYFP fusion protein constructs. The six *LHCP* gene CDSs, *FtsY* gene CDS, *LTD* gene CDS, and *cpSRP54* gene CDS without stop codon were cloned to pSPYCE(M) through BamHI and SmaI sites to produce cYFP fusion protein constructs. The primer pairs used for pSPYNE(R)155 and pSPYCE(M) vector construction are listed in Supplementary Data Table S8. The 10 fusion protein constructs were transformed into *A. tumefaciens* strain GV3101. Then the pSPYCE(M) fusion plasmid and pSPYNE(R)155 fusion plasmids were infiltrated into *Nicotiana benthamiana* tobacco leaves. After 60 hours at 26°C, the epidermis of tobacco was observed under a laser scanning confocal microscope (Leica TCS-SP8 SR, Germany). The YFP fluorescent signals were detected with a 514-nm excitation wavelength.

### Statistical analysis

Data from this study were subjected to statistical analysis and significance tests were performed using one-way ANOVA with Duncan’s multiple range test (*P* < .05). An asterisk indicates a significant difference in the data, and the data presented were analyzed by SPSS 22.0 (Statistical Package for the Social Sciences, Chicago, IL, USA) on a Windows PC. The histograms were generated using GraphPad Prism 6 (GraphPad Software Inc., San Diego, USA), and the mean ± standard deviation is represented with bar graphs.

## Supplementary Material

Web_Material_uhac212

## Data Availability

Data are available upon request to the corresponding author.
