# Supplementary material for: Identification and characterization of CsSRP43, a major gene controlling leaf yellowing in cucumber
Source: Hortic Res. 2022 Dec 1;9:uhac212. doi: 10.1093/hr/uhac212 (PMC9719040; doi:10.1093/hr/uhac212)
Supplement: Web_Material_uhac212 [file web_material_uhac212.zip › Supplemental figures.doc]

Fig.S1 The number of chloroplast in the leaves cells of the Gy14 and *yf*. Data were represented as means ± SD of seven replicates.

Fig.S2 Expression analysis of five candidate genes in Gy14 and *yf*. Data were represented as means ± SD of four replicates.

**A**

**B**

**C**

Fig.S3 GO enrichment analysis of the DEGs between *srp43-1* and WT. A The biological process categorys. B The cellular component categorys. C The molecular function categorys
